# Silencing Nuclear Pore Protein Tpr Elicits a Senescent-Like Phenotype in Cancer Cells

**DOI:** 10.1371/journal.pone.0022423

**Published:** 2011-07-19

**Authors:** Brigitte David-Watine

**Affiliations:** Institut Pasteur, CNRS URA2582, Groupe E3 Biologie Cellulaire du Noyau, Paris, France; Texas A&M University, United States of America

## Abstract

**Background:**

Tpr is a large coiled-coil protein located in the nuclear basket of the nuclear pore complex for which many different functions were proposed from yeast to human.

**Methodology/Principal Findings:**

Here we show that depletion of Tpr by RNA interference triggers G0–G1 arrest and ultimately induces a senescent-like phenotype dependent on the presence of p53. We also found that Tpr depletion impairs the NES [nuclear export sequence]-dependent nuclear export of proteins and causes partial co-depletion of Nup153. In addition Tpr depletion impacts on level and function of the SUMO-protease SENP2 thus affecting SUMOylation regulation at the nuclear pore and overall SUMOylation in the cell.

**Conclusions:**

Our data for the first time provide evidence that a nuclear pore component plays a role in controlling cellular senescence. Our findings also point to new roles for Tpr in the regulation of SUMO-1 conjugation at the nuclear pore and directly confirm Tpr involvement in the nuclear export of NES-proteins.

## Introduction

Nuclear pore complexes (NPCs) mediate all selective bidirectional transport between the nucleus and the cytoplasm. Evidence is also emerging pointing to additional roles for NPCs, their constituent proteins (nucleoporins) and associated transport proteins, some of which are independent of classical transport [1], [2]. It may therefore reasonably hypothesized that NPCs are key in pathological cell conditions where abnormal cell growth is a central feature.

The protein Tpr (for translocated promoter region) and its homologues are a conserved component at the nuclear side of NPC. In mammals Tpr is a 267-kDa structural protein with a long N-terminal domain that associates in a dimer to form a parallel, two-stranded coiled-coil. The C-terminal domain is highly acidic and is predicted to be unstructured [3]. In mammalian cells Tpr is restricted to the nucleoplasmic fibrils of the NPC and it has been suggested that it acts as the main architectural element of the nuclear basket [4], [5]. Mammalian Tpr is tethered to NPC through interactions with Nup153 [5], [6], [7], [8]. Many functions have been attributed to Tpr and its homologues in different species in addition to a role in NPC architecture. These include mRNA export control [9], [10], nuclear protein export [4], [11], silent telomeric chromatin organization and telomere length control [12], [13], [14]. In addition, Drosophila and human Tpr have been shown to be involved in spindle checkpoint control [15], [16], [17] and human Tpr in the control of Erk2 nucleo-cytoplasmic translocation [18].

Tpr homologues in yeast, Mlp1p-Mlp2p, are involved in attaching SUMO-protease Ulp1p to NPC [19], [20]. This functional interaction seems to be conserved in Arabidopsis thaliana between ESD4 and NUA, which are homologues of Ulp1p and Tpr, respectively [21]. SUMOylation corresponds to the post-translational conjugation of SUMO (small ubiquitin-related modifier protein) to a specific lysine residue in a target protein. SUMOylation is mediated by an enzymatic cascade reaction involving successively a SUMO-activating enzyme or E1 and a unique E2 SUMO-conjugating enzyme, Ubc9. SUMO modification is reversible since SUMO-specific proteases can deconjugate the SUMO moiety from the modified proteins suggesting that protein SUMOylation is dynamically regulated in cells [22], [23]. Among the Ulp1p-related SUMO-proteases that have been characterized in mammals, SENP1 and SENP2 show the greatest similarity with yeast Ulp1 and are also associated with the nuclear envelope. Ulp1p and SENP2 share the property of being targeted at NPC by their non-catalytic N-terminal domains [24]. However, in vertebrates, the N-terminal NPC-targeting domain in SENP2 interacts with the C-terminal domain in Nup153, but not with Tpr [25], [26].

Cellular senescence was first described as “replicative senescence” because of the limited life span of human diploid fibroblasts in vitro [27]. Studies in cancer cells have shown that, despite the fact that they are able to divide indefinitely, a state closely resembling replicative senescence can be acutely induced by various stimuli such as chemotherapeutic agents and radiation. This therefore represents another cell program, besides apoptosis, that can limit cell proliferation [28], [29]. Senescent cells are characterized by enlarged cell size, flattened morphology, inability to synthesize DNA, and expression of senescence-associated (SA)-β-galactosidase, the biomarker of senescence [30].

We report here that Tpr is critical for cell proliferation and that Tpr depletion leads to a senescence-like phenotype that is dependent on p53. Also, Tpr down-regulation impacts on the nuclear export of proteins with a Crm1-dependent nuclear export sequence (NES). Levels of Nup153 and SENP2 function at the nuclear pore are also affected. As a consequence substantial changes were observed in the pattern of SUMO-1 conjugation at NPC and within the cell.

## Results

### Tpr knockdown induces G0–G1 arrest and a senescent phenotype in HeLa cells

**Figure 1:**
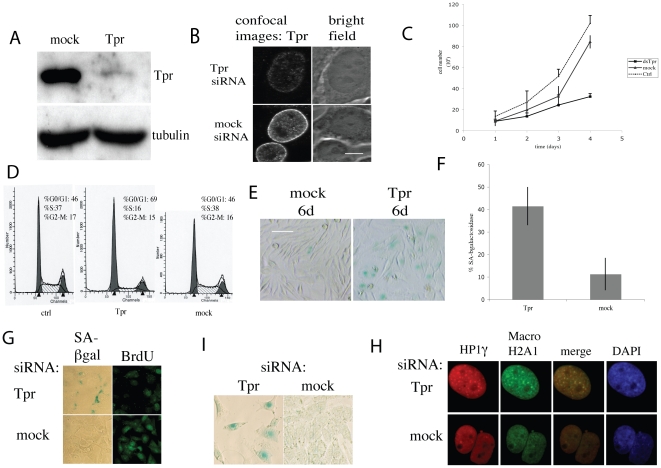
Cell cycle effects of Tpr depletion in HeLa cells. HeLa cells seeded in 24-well plates at 5 10^−4^ cells per well were treated with Tpr or mock siRNAs. 1A. 48 hours after siRNA treatment as indicated on top of the figure, a cell extract was prepared and analyzed by western blotting using anti-Tpr Mab 203-37; tubulin was used as loading control. 1B. Bright field image (right panel) and confocal image (left panel) of HeLa cells labeled with an anti-Tpr monoclonal antibody after 2 days of transfection with Tpr or mock siRNA. Scale bar: 5 µm. 1C. Growth curve of HeLa cells transfected with Tpr, mock siRNA or not transfected: control (Ctrl). 1D. Representative FACS profiles of mock siRNA treated (left panel), Tpr siRNAs treated cells (center) and non-transfected cells or control (right). Ethanol-fixed cells were incubated with PI and analyzed by FACS for ploidy. Cell number is represented on the vertical axis and DNA content on the horizontal axis. G0–G1 and G2-M phases are represented as first and second peaks starting from the vertical axis, respectively. The intermediate striped domain corresponds to the S phase. 1E. Induction of senescence: cells were plated at low density and treated with Tpr or mock siRNAs. After 6 days, cells were fixed and stained for SA-β-gal activity at pH 6.0. Scale bar: 20 µm. 1F. Quantification of SA-β-gal positive cells in cultures depleted of Tpr and stained for SA-β-gal activity at pH 6.0. 1G–1H. U2OS cells were transfected with Tpr or mock siRNAs. 1G: After 6 days the cells were fixed and stained for BrdU incorporation and SA-β-gal activity at pH 6.0. Scale bar: 15 µm. 1H: After 6 days, the cells were labeled with anti-HP1γ and anti-MacroH2A antibodies and DAPI. Scale bar: 5 µm. 1I: A375 cells were transfected with Tpr or mock siRNAs. After 6 days the cells were fixed and stained for SA-β-gal activity at pH 6.0. Scale bar: 15 µm.

Tpr has recently been described to have several different functions but its role in cell cycle, cell fate and proliferation has never been fully assessed. We thus investigated the effect of Tpr depletion on the cell cycle using siRNAs targeting Tpr in HeLa cells. We found that the level of Tpr protein was specifically reduced after 48 hours of treatment with either of the two synthetic siRNAs used, as shown by western blot analysis of total cell extracts of treated HeLa cells (Fig. 1A and S1A).

We then determined the location in HeLa cells by confocal microscopy (Fig. 1B). Optical sections through the center of the nucleus showed the punctuate pattern characteristic of nucleoporins at the NE. Exposure to siRNAs directed against Tpr dramatically reduced the punctuate Tpr pattern at the NE (Fig. 1B). Since Tpr was efficiently down regulated under these conditions, we were able to analyze its role in cell growth and viability. We found that treatment with Tpr siRNAs impaired cell proliferation as compared to mock siRNA-treated cells (Fig. 1C). To investigate whether this was due to halted cell proliferation or cell death, cells were treated with Annexin-V and propidium iodide to label the nuclei of dead cells and the cells were analyzed by fluorescence-activated cell sorting (FACS). No gross difference between the Tpr siRNA-treated cells and the controls was observed, indicating that the growth defect was not due to apoptosis (data not shown).

To further characterize the effect of Tpr knockdown on cell proliferation, we analyzed cell cycle distribution of Tpr siRNA-treated cells and controls by measuring their DNA content by FACS. We noted that mock siRNA transfection did not affect the cell cycle of HeLa cells as compared to untreated cells. By contrast, the cell cycle was arrested at the G0–G1 phase in Tpr knockdown cells as shown in Fig. 1D. Approximately 46% of the control transfected and untransfected cells (2 days post-transfection) were in the G0–G1 phase of the cell cycle, whereas 69% of the Tpr knockdown cells were in G0–G1. At the same time, approximately 37–38% of control cells progressed to the S phase compared with only 16% of the Tpr knockdown cells (Fig. 1D). The same proportion of cells were seen in the G2-M phase under all three conditions. Similar results were obtained with the two Tpr siRNA sequences (Fig. S1B).

We then performed a colony-forming assay as an independent measurement of the proliferation defect triggered by Tpr depletion. A substantial decrease (36%) in the number of colonies was apparent when cells were transfected with Tpr siRNAs compared to mock-transfected cells (Fig. S1C). These results suggest that transfecting HeLa cells with Tpr siRNAs dramatically slowed tumor cells proliferation.

A more careful examination of the Tpr siRNA-treated cells showed that a fraction of these had a rather “severe” phenotype in that they had the appearance of senescence with a grossly expanded cytoplasm. This led us to conduct further investigations focused on this particular response. In transient RNAi assays, the time course for effective depletion of the target protein is not more than four days while Tpr knockdown cells were arrested in G0–G1. Meanwhile non-transfected cells continue to divide and rapidly overtake non-dividing cells. We therefore established conditions under which cells were plated at low density (5.10^4^ to 10^5^ per well in a 6 well-plate) to minimize the effect of overgrowing non-transfected cells.

Experiments under these conditions showed that a large fraction of the population started to change morphology 3–4 days after transfection: cells were enlarged and flattened (Fig. 1E). A major test for senescence is SA-β-galactosidase activity measured at acidic pH [30] and 6 days post-transfection, most of the enlarged and flattened cells were β-gal positive and colored blue (about 40%) in Tpr knockdown wells, whereas senescent-like blue cells were rare (around 10%) in the mock treated cell (Fig. 1F).

Tpr siRNA knockdown also induced senescence in two other tumor cell lines, U2OS, a human osteosarcoma cell line, and A375 human melanoma cells (Fig. 1G–1I). BrdU staining showed that SA-β-galactosidase positive cells were BrdU negative (Fig. 1G). In addition, Tpr siRNAs-treated U2OS cells and controls were labeled with antibodies specific for histone variant macro-H2A and HP1γ, two markers of heterochromatin constitutive of the SAHF (senescence associated heterochromatin foci) that were first identified in senescent human fibroblasts [31], [32]. Many macro-H2A and HP1γ positive and colocalizing foci were observed in U2OS senescent cells nuclei, whereas they were absent in control nuclei. Brighter DAPI dots indicating heterochromatin colocalized with macro-H2A and HP1γ labeling can be seen in Fig. 1H.

### Tpr depletion induces nuclear accumulation of p53

As the p53 pathway is often involved in blocking tumor development by triggering cellular senescence or apoptosis in response to stress it was important to evaluate cell p53 status when Tpr is depleted.

**Figure 2:**
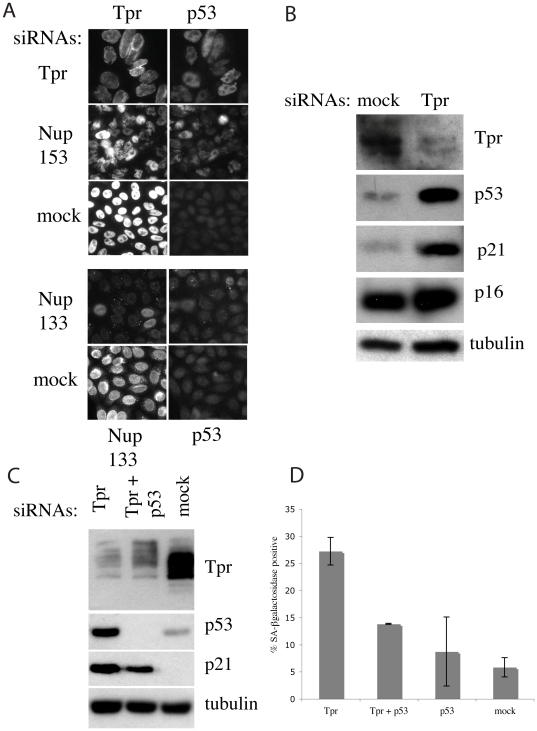
p53 is stabilized and accumulates in the nuclei of Tpr-depleted cells. 2A. Analysis of p53 distribution in HeLa cells by immunofluorescence. Upper panel: HeLa cells were transfected with Tpr, Nup153 and mock siRNAs as indicated on the left; lower panel: HeLa cells were transfected with Nup133 and mock siRNAs. The cells were fixed at day 2 post -transfection and labeled with an anti-Tpr antibody (top left panels) or anti-Nup133 (bottom left panel) and an anti-p53 (right top and bottom panels). 2B. Hela cell whole cell protein lysates were separated by SDS-PAGE and immunoblotted with antibodies specific for Tpr, p53, p21, p16 and tubulin as indicated in the figure. 2C. Whole cell protein lysates of U2OS cells treated with Tpr, Tpr+p53 or control (mock) siRNAs were separated by SDS-PAGE and immunoblotted with antibodies specific for Tpr, p53, p21 and tubulin as indicated in the figure. 2D. Co-depletion of p53 and Tpr reversed senescence induction. Cells were transfected with mock, Tpr, p53 or Tpr and p53 in combinations as indicated. The final siRNA concentration was 100 nM in each case and compensation was made with mock siRNAs. Cells were labeled for SA-β-galactosidase at 6 days post-transfection and the result is given as the percentage of SA-β-galactosidase positive cells in each of the three independent experiments.

We first used immunocytochemistry to analyze the distribution of p53 and at the same time studied the effect of Nup153 depletion as this has been shown to delocalize Tpr from the nuclear pore to the nuclear interior. As shown in Fig. 2A, the p53 signal appeared as intense punctuate labeling in the nuclei of Tpr- and Nup153- depleted cells. No signal was observed in control cells. By comparison, whereas depletion of Nup133, a scaffold nucleoporin that plays an important role in nuclear pore structure and function, does not induce any nuclear accumulation of p53 [33], such accumulation was seen in Tpr-depleted U2OS cells (data not shown). These results show that Tpr depletion or mislocalization lead to nuclear accumulation of p53.

The cyclin-dependent kinase inhibitor (CDK) p21 is elevated in many stressful conditions and is transcriptionally regulated by p53. We therefore investigated the consequences of Tpr depletion on levels of p53 and p21. Levels of p53 and p21 were both up-regulated in Tpr-depleted Hela cells (Fig. 2B) and U2OS cells (Fig. 2C). In addition, when evaluated in cell extracts where both Tpr and p53 were depleted, p21 accumulation was seen to be lower than with Tpr siRNAs alone (Fig. 2C). This observation indicates that part of the p21 accumulation noted is due to activation of p53. Levels of the CDK inhibitors p16^INKA^ (Fig. 2B) and cyclinD1 were also increased in Tpr-depleted HeLa cells (data not shown).

Finally, in order to demonstrate formally the role played by p53 in mediating the induction of senescence in Tpr-depleted cells, we co-depleted both Tpr and p53. Knocking down both Tpr and p53 resulted in a substantial reversion of the senescence phenotype as assessed by the percentage of SA-β-galactosidase-positive cells (Fig. 2D).

### Tpr depletion interferes with NES-dependent nuclear export

It has been suggested that the low steady-state levels of p53 in normal cells, i.e. in the absence of cellular stress, result from continuous Crm1-dependent nuclear export [34] and subsequent cytosolic degradation of p53. Also, Tpr was recently found to interact with Crm1 in an trimeric export complex [11]. We may therefore hypothesize that Tpr leads to p53 nuclear accumulation and stabilization by blocking its nuclear export.

**Figure 3:**
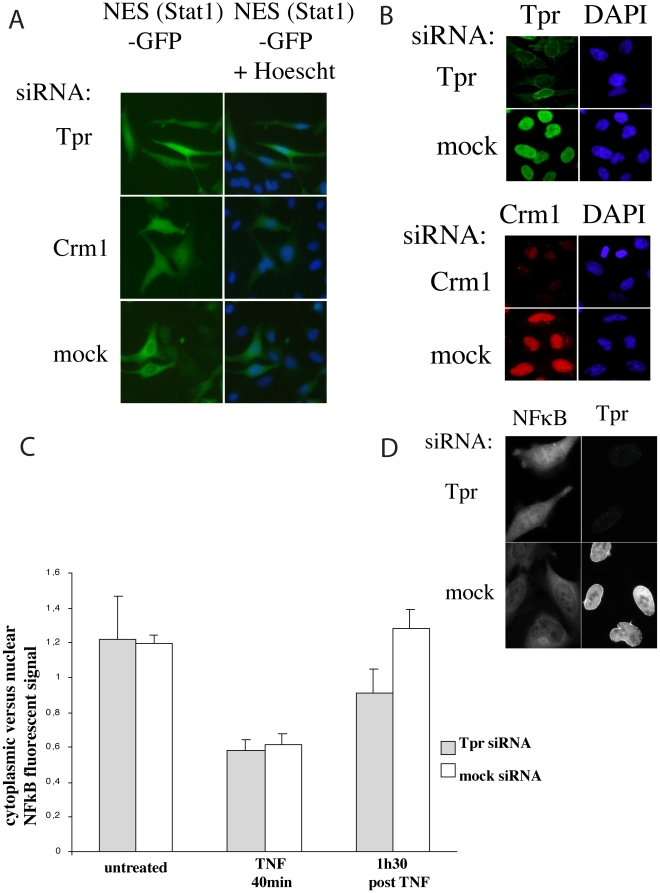
Analysis of Crm1 dependent NES-proteins nuclear export in Tpr depleted cells. 3A–3B. Distribution of GFP in cells co-transfected with STAT1-NES-GFP and Tpr, Crm1 or control (mock) siRNAs. 3A: GFP distribution was analyzed in live cells. Nuclei were labeled with Hoechst 33342. Scale bar: 10 µm. 3B: Cells were then permeabilized and labeled with antibodies specific for Tpr and Crm1 and DAPI for the DNA. Top panel: Tpr depletion and control; lower panel: Crm1 depletion and control. Scale bar: 10 µm. 3C–3D: Tpr depletion delays nuclear export of NFκB. HeLa cells were transfected with Tpr or mock siRNAs 2 days prior to NFκB induction. Transfected and control cells were then incubated with TNF 100 IU/mL for 40 min. TNF was then removed from the medium and the cells were either fixed or kept for another 1h30 in fresh medium before fixation. 3C: Quantification of the NFκB signal: All images were acquired at the same magnification and exposure and the NFκB signal was quantified using the same surface area in the nuclei and cytoplasm of cells under the different experimental conditions using OpenLab3.1.2. The cytosolic signal was plotted against the nuclear signal and standard deviation was calculated using Microsoft Excel. Error bars represent the standard deviation. Note that the cytosolic to nuclear signal ratio is very similar in control cells before TNF treatment and 1h30 after TNF removal. This was as expected and is due to NFκB fully returning to the cytoplasm at this time. 1h30 after TNF removal the cytosolic to nuclear signal ratio was about 25% lower in the Tpr-depleted cells than in the control cells. 3D: Immunofluorescent labeling of NFκB and Tpr 1h30 after TNF removal in Tpr-depleted and control cells. Fixed cells were labeled with a rabbit anti-NFκB antibody and anti-Tpr mAb 203-37.

To test this hypothesis we first used the construct pSTAT1-NES-GFP, which encodes residues 367–427 of human STAT1 that confers NES activity [35], to prevent additional regulatory events interfering with the inhibition of Crm1-dependent nuclear export. This construct was co-transfected with Tpr, Crm1 and control siRNAs to probe for NES-dependent export. As shown in Fig. 3A, the distribution of the GFP signal in live cells 48 hours after transfection showed that Crm1- and Tpr-depletion led to substantial nuclear retention of the NES-GFP construct whereas GFP remained mainly cytosolic in mock-treated cells. The cells were then permeabilized and labeled with Crm1 and Tpr antibodies to check depletion efficiency (Fig. 3B).

A functional Crm1-dependent NES is also required for IkappaBepsilon-mediated nuclear export that controls the nucleo-cytoplasmic distribution and post-induction nuclear clearance of NFκB/Rel proteins [36]. To better assess the effect of Tpr depletion on the NES-nuclear export of an endogeneous protein, HeLa cells were first transfected with Tpr or mock siRNAs two days prior to NFκB induction by TNF. As shown in Fig. 3C–3D, Tpr depletion delayed the clearance of NFκB from the nucleus as compared to mock treated cells. It is worth noting that NFκB accumulation in the nucleus following TNF treatment was not different in mock- and Tpr-siRNAS treated cells (Fig. 3C).

We then sought to determine whether Tpr depletion affects Crm1 levels and distribution, or vice-versa. To do this we transfected HeLa cells with siRNAs directed against Tpr and, Crm1, or mock transfected HeLa cells, and 48 hours later we treated the cells with Tpr- and Crm1-specific antibodies. Immunofluorescent analysis for Crm1 expression in control and Crm1 knock-down cells showed reduced levels in the nucleoplasm and nuclear envelope of the Crm1 siRNA-transfected cells. Also Tpr expression was substantially reduced in Tpr siRNAs-treated cells. By contrast, Crm1 expression did not appear to be affected by Tpr depletion and Tpr was not affected in Crm1 knock-down cells (Fig. S2A–B).

### Tpr depletion impacts on the expression and distribution of Nup153 and SENP2 in the nuclear basket

**Figure 4:**
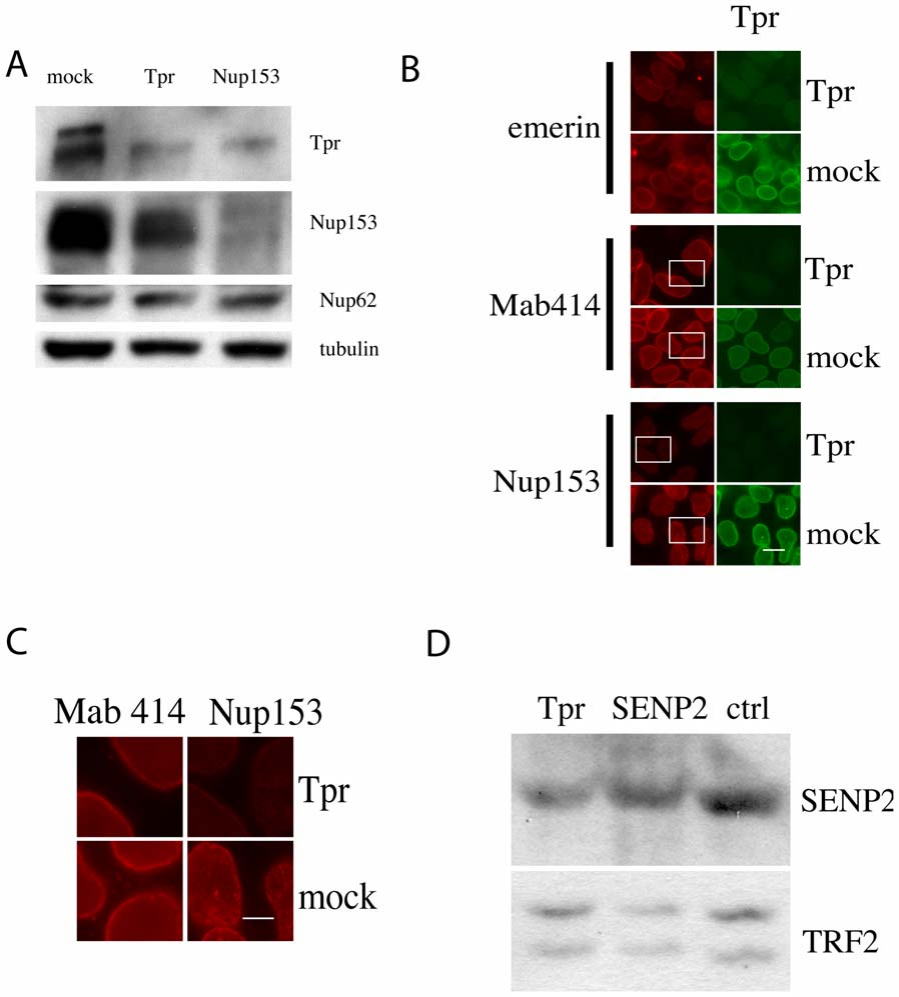
Analysis of Nup153 and SENP2 proteins in Tpr-depleted cells. 4A. HeLa cells were transfected with mock, Tpr or Nup153 siRNAs as indicated on the top. Whole cell lysates were analyzed 2 days post-transfection by immunoblotting using antibodies directed against Tpr, the anti-FG repeat nucleoporin Mab414 to label Nup153 and Nup62 and anti-tubulin as loading control. 4B–4C. Nup153 expression is specifically lowered in Tpr-depleted cells. HeLa cells were treated with Tpr2 or mock siRNAs for 2 days. 4C. Cells were fixed and labeled with an anti-Tpr antibody and anti-emerin, anti-Mab414 or anti-Nup153 antibodies. Scale bar: 15 µm. 4D. Details from Mab414 and Nup153 labeling. Scale bar: 5 µm. 4D. Analysis of SENP2 expression in nuclear extracts of 293 cells treated with Tpr, SENP2 and mock siRNAs by western blot analysis. TRFII is used as a loading control.

To better assess the role of Tpr in nuclear pore organization and function, we examined more carefully the relationship between Tpr and Nup153 by measuring their levels in total extracts of cells transfected with Tpr or Nup153 siRNAs. As shown by western blot analysis of total cell extracts (Fig. 4A), cell treatment with Nup153 siRNAs led to almost complete disappearance of Nup153 and a marked decrease in Tpr. This decrease in Tpr levels was consistent with previous studies showing that Tpr is mislocalized to the nuclear interior in Nup153 knockdown cells [8]. Likewise, treatment with Tpr siRNAs led to a marked decrease in Tpr and a greatly reduced Nup153 signal (Fig. 4A). We next confirmed this observation by immunofluorescence analysis in HeLa cells treated with Tpr siRNAs and labeled with antibodies directed against different components of the nuclear envelope. Labeling with an anti-emerin antibody and the Mab414 antibody that recognizes all FG-repeat-containing nucleoporins including Nup153 was not grossly modified (Fig. 4B–4C). By contrast, we found that labeling with a specific anti-Nup153 antibody was reduced (Fig. 4B–4C). We concluded from these observations that Tpr depletion specifically affects level of Nup153 protein at the NE.

Given that Tpr depletion altered Nup153 distribution at the nuclear pore and that Nup153 tethers SENP2 to the NPC, we hypothesized that Tpr may impact on SENP2 protein levels and function. To test this hypothesis, we evaluated the effect of Tpr depletion on SENP2. Firstly, given that no antibody is available to observe endogenous SENP2 in fixed cells, we analyzed SENP2 levels in nuclear extracts of cells depleted in Tpr, Nup153 or SENP2 and in mock depleted cells. Tpr depletion in HeLa and 293 cells was accompanied by a reduction in SENP2 to levels very similar to those observed in SENP2-depleted extracts. Then to compare the different protein levels, we monitored Nup133 and TRF2 as a loading control for nuclear extracts (Fig. 4D and S3A). Depletion of Nup153 similarly induced a reduction in SENP2 (data not shown).

### Tpr depletion affects SUMOylation

**Figure 5:**
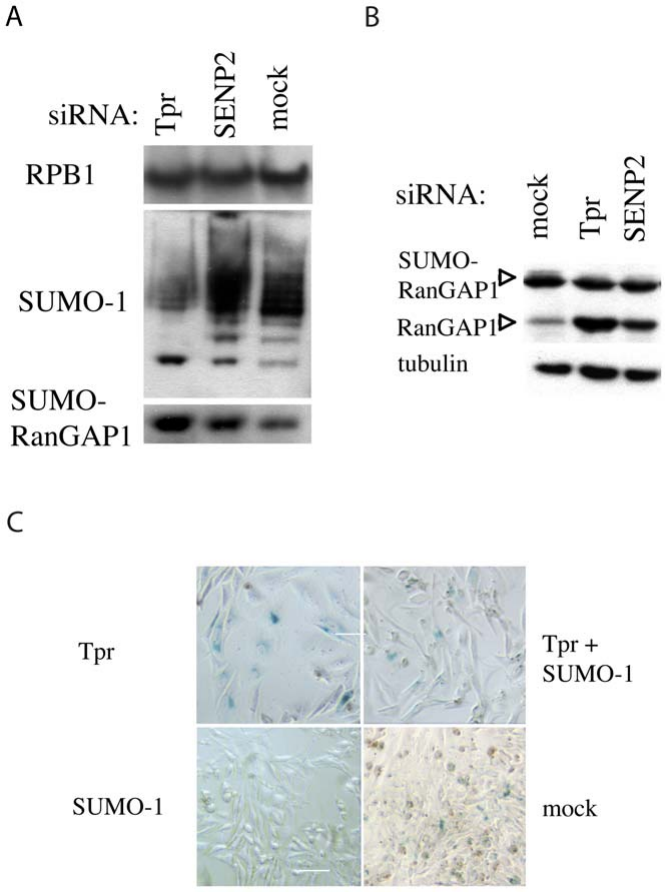
Tpr regulates SUMOylation. 5A–5B. Analysis of the overall level of SUMOylation in cells transfected with Tpr, Nup153, SENP2 and mock siRNAs. 5A. Nuclear extracts of siRNAs treated cells were prepared and analyzed by western blotting using the antibodies as described on the left of the figure. RPB1 was used as loading control. 5B. Analysis of RanGAP1 SUMOylation. Cytoplasmic extracts of Tpr, Nup153, SENP2 and mock siRNAs-treated cells were prepared and analyzed by western blotting using an anti-RanGAP1 antibody recognizing both SUMOylated and non-SUMOylated forms of RanGAP1 and with tubulin as loading control. 5C: SUMO-1 siRNA treatment overrides Tpr depletion-induced senescence in HeLa cells. HeLa cells were transfected with Tpr, SUMO-1, Tpr and SUMO-1 and mock siRNAs. After 6days, the cells were fixed and stained for SA-β-gal activity at pH 6.0. A representative picture of each condition is presented. Scale bar: 15 µm.

The finding that Tpr depletion affects SENP2 prompted us to address the question of whether Tpr depletion might interfere with the SUMOylation of other proteins. In order to investigate changes in the overall SUMOylation of proteins, HeLa cells were treated with Tpr, SENP2 and control siRNAs and nuclear and cytoplasmic extracts were analyzed and compared by western blotting using an anti-SUMO-1 antibody. Blots of nuclear extracts showed that SENP2 repression caused an accumulation of high molecular weight SUMO-1 as was expected for a decrease in SUMO-protease activity. No such accumulation was seen in mock-transfected cells. By contrast, Tpr-depleted cells showed lower overall SUMOylation than compared siRNAs transfected cells (Fig. 5A). Similar results were obtained in U2OS and 293 cells (Fig. S3B and data not shown). Consequently, free SUMO-1 was seen to accumulate in Tpr-depleted cells (Fig. S3C).

RanGAP1 (Ran-GTPase activating enzyme 1) is an abundantly SUMOylated protein and most anti-RanGAP1 antibodies detect the SUMO-1-modified form and the SUMO-free RanGAP1, migrating around 80 kDa and 70 kDa, respectively. We thus examined the effect of Tpr depletion on the distribution and the level of expression and SUMOylation of RanGAP1. The fraction of SUMO-free RanGAP1 detected by this antibody was increased in cytosolic extracts of Tpr- and SENP2-siRNAs treated compared to mock treated HeLa cells (Fig. 5B) and in U2OS whole cell extracts (Fig. S3B). In the nuclear extract fraction, only SUMO-1-RanGAP1 was detected and found to be increased in Tpr- and SENP2-siRNAs treated cells compared to mock treated cells when detected with anti-SUMO-1 and anti-RanGAP1 antibodies, with the increase being more pronounced in Tpr-treated cells than in SENP2-treated cells (Fig. 5A).

So far, the role of protein SUMOylation in senescence induction has only been studied in normal fibroblasts. We have verified that depletion of SENP2 could induce senescence in U2OS cells in our experimental setting (Fig. S3D). To better assess the contribution of the SUMO-1 modification pathway to the Tpr-induced senescence phenotype in cancer cells, we performed in parallel RNAi knockdown of Tpr, SUMO-1 and combined SUMO-1 and Tpr. After 6 days, cells were fixed and analyzed for SA-β-galactosidase staining. The total cell number was dramatically different between the Tpr alone condition and the two other conditions. Forty or 50 fields were counted for each condition: the number of fields containing blue cells and the percentage of space occupied by enlarged blue cells were evaluated (Table 1). In fact, for SUMO-1 depleted cells, most fields were filled with cells at saturation density meaning that the cell proliferation was not stopped. Very few blue cells were observed: 3 (in one field) to about 20 blue cells or so could be enumerated in 7 fields only, the rest being negative. In the end, the results were so different that it was impossible to include them in Table 1. As a matter of fact there were less SA-β-galactosidase positive cells in the SUMO-1 depleted cells than in the mock control. The results described in Table 1 show that the population as a whole was affected by Tpr depletion whereas cells with a senescent-like phenotype were far more dispersed in the Tpr plus SUMO-1 condition. In addition, with few exceptions, most blue cells did not exhibit a full senescent phenotype as they were not flattened and the SA-β-galactosidase staining was faint (Fig. 5C).

**Table 1:** Effects of SUMO-1 depletion on Tpr-induced senescence.

SABS (%)	0	5–10	25–40	>40
Tpr	a	19 (4)	45 (13)	34 (16)
Tpr+SUMO-1	59 (6)	37 (6)	a	0

After 6 days cells transfected with Tpr or Tpr and SUMO-1, as described in Fig. 5C, were analyzed for SA-β-galactosidase staining. Forty to fifty fields were counted for each condition: the number of fields containing blue cells and the percentage of space occupied in each field by enlarged blue cells were evaluated. SABS: percentage of space occupied by blue cells in a field. Results are given as percentages of fields in the four categories for Tpr or Tpr+SUMO-1 experiments. Numbers between round brackets correspond to standard deviation. (a): only one field with blue cells was observed.

## Discussion

We have demonstrated in this study that depleting Tpr is sufficient to induce a senescent-like phenotype in tumor cell lines. The senescence phenotype can be explained by the accumulation of several growth-suppressive proteins acting on mitogenic signal transduction and cell cycle regulation. Two main signaling pathways, p16INK4a–pRb and p14ARF–p53, are responsible for the execution of proliferative arrest that characterizes senescence [37], [38]. The Rb-p16 pathway is defective in both HeLa and U2OS cell lines but p53 in U2OS and HeLa cells and p16 in HeLa cells are increased after Tpr knockdown. P53 accumulates in the nucleus and so does p21/CIP1, a downstream effector that is involved in several forms of G1 checkpoint control. Furthermore, part of the p21/Cip1protein up-regulation is dependent on the presence of p53.

In normal cells, p53 is a target of the ubiquitin-proteasome pathway: the low levels of p53 result from continuous Mdm2-mediated nuclear export of p53 for cytosolic degradation. In HeLa cells, nuclear export is also necessary for degradation of p53 mediated by the human papilloma virus (HPV) 18 E6 protein [39]. We show here that Tpr depletion leads to nuclear retention of a GFP reporter harboring the NES sequence of STAT1 and delays the post-induction nuclear clearance of NFκB after TNF removal in HeLa cells [36], two events dependent upon the Crm1 export factor. Tpr was recently found to interact with Crm1 in the presence of an NES peptide or an NES peptide plus RanGTP like in a trimeric export complex [11]. Although the molecular details of this interaction have not been further characterized, the absence of Tpr is likely to impair the function of Crm1 in nuclear export. We found that Tpr knock-down weakly affects Crm1 levels in western blot experiments but does not obviously impact on Crm1 levels as measured using specific antibodies on fixed cells. However, it has previously been reported that even slight downregulation of Crm1 results in prominent defects in nuclear export [40]. Crm1 inhibition also reduced cell proliferation and induced profound cell alterations and apoptosis (data not shown), as previously reported [41]. We thus favor the hypothesis that a main consequence of Tpr depletion is the nuclear accumulation and activation of p53 by inhibition of its nuclear export.

In support of this, leptomycin B (LMB), a Crm1 inhibitor, has been shown to reduce E6's ability to degrade p53 in cervical carcinoma cells [39], [42] and cause: (i) retention of STAT1-NES construct in the nucleus [34], [35], (ii) nuclear accumulation and activation of p53, (iii) induction of specific downstream target genes of p53 [42]–[45] and (iv) growth arrest and a senescent-like phenotype primary prostate epithelial cells and fibroblasts.

Consistently with this, we show here that nuclear accumulation of p53 is accompanied by growth arrest and senescence in HeLa, U2OS and A375 cells, as characterized by enlarged and flattened SA-β-galactosidase-positive and BrdU-negative cells. Chromatin foci were labeled with HP1γ and macro-H2A antibodies, two markers of SAHF, in the nuclei of senescent U2OS cells [31], [32]. Finally, the induction of senescence is substantially alleviated when p53 is co-depleted with Tpr.

We also reported that Tpr depletion induces a marked decrease in Nup153 total cell content and at the NPC. In addition to causing a nuclear redistribution of Tpr [8], Nup153 knockdown is accompanied by a decrease in total cell Tpr content (this paper). Fluorescence recovery after photo bleaching measurements of the association of Nup153 with NPCs showed that Nup153 exists as a fast and a slower exchanging pool [46] and it has been suggested that the slower fraction may anchor Tpr at the nuclear basket [47].

Indeed, the effect of Tpr depletion on Nup153 is partial, which may explain why the observation in fixed cells required substantial Tpr depletion. Only a fraction of the Nup153 was affected by this Tpr depletion whereas Nup153 depletion has more profound effects on Tpr. The mechanism by which the depletion of one protein leads to at least partial degradation of the other partner has not yet been analyzed.

Tpr depletion was expected to impinge on SENP2 as this interacts directly with Nup153 in human cells [25], [26]. We observed that SENP2 levels decreased in Tpr-siRNAs treated cells in the same range as in SENP2-siRNAs treated cells. However, whereas siRNA-mediated depletion of SENP2 led to an accumulation of high-molecular-mass SUMO-1-modified species, consistent with decreased SUMO-protease activity, Tpr depletion had the opposite effect with decreased levels of high-molecular-mass SUMO-1-modified species. SUMOylation is a highly dynamic post-translational modification, and loss of SUMOylation may result either from decreased SUMO conjugating activity or increased SUMO protease activity.

SENP2 is a shuttling, predominantly nuclear, protein and depends on Crm1 for its nuclear export [48]. The importance of NPC localization for the correct regulation of SENP2 activity has already been emphasized as mutants of the NLS region - the interaction region with Nup153- are unable to locate at the nuclear pore and they show enhanced SUMO-protease activity [25]. Therefore the changes in SUMOylation may be attributed to altered SENP2 localization and regulation in the absence of Tpr, presumably with SENP2 being retained in the nucleus as a Crm1-export dependent protein. Altered SENP2 regulation may have several consequences. Firstly, selective modifications of proteins by either SUMO-1 or SUMO-2/3 [49], [50] affect their sub-cellular distribution and function [49], [50]. Notably, SUMO-1 is distributed only to NPCs [51] and to the mitotic spindle during mitosis [52], and altered localization and function of different checkpoint proteins was described during mitosis in Tpr-depleted cells [15], [16], [17]. Secondly, it was recently proposed that selective modification of RanGAP1 and probably other targets by SUMO-1 or SUMO-2/3 is determined at the deconjugation level by SUMO-proteases [53]. As a consequence an increase in SENP2 activity could induce a shift in the spectrum of SUMO conjugation from SUMO-1 to SUMO 2/3 in Tpr-depleted cells. Interestingly, it has been reported that the stable overexpression of SUMO 2/3 induces a senescent phenotype in 293 cells, probably by mimicking the stress-induced elevation of SUMO 2/3 [54].

Surprisingly, Tpr depletion and SENP2 have similar effects on RanGAP1, with increased amounts of SUMO-free RanGAP1 in the cytoplasm and SUMO-1-RanGAP1 at the nuclear envelope. An increase in total RanGAP1 has previously been reported in conditions of cellular stress [55], [56]. In SENP2 depleted cells, higher levels of SUMO-1 conjugation may render free SUMO-1 less available for conjugation in the vicinity of the nucleus, thus affecting the SUMOylation of cytosolic RanGAP1, as previously observed [57].

Tpr depletion has a direct impact on the nuclear export of proteins [4], [11] (this paper). Therefore, Tpr may play a complementary role in protein retention in the nucleus, acting in concert with SENP2, the impairment of which may result in altered function and sub cellular localization of particular targets, notably transport factors, nucleoporins and transcription factors. In addition, it is conceivable that Tpr, Nup153 and SENP2, given their interdependency, are organized into a complex whose function is modified when any of its constituents are depleted.

Altogether, our data underline the evolutionary conservation of the role of Tpr, in association with Nup153, in docking a SUMO protease and regulating SUMO conjugation at the NPC [19], [20], [24]. Initially, the association of E3 ligases and isopeptidases with NPCs suggested that import and export were coupled with modification and de-modification, respectively [58]. SUMOylation processes are now known to be associated with other roles for NPCs including cell division, DNA repair, DNA replication and mRNA quality control. More recently, a systematic genomics approach aimed at evaluating the relationship between the SUMO pathway and functionally associated proteins in yeast, identified many components of the NPC and associated factors, notably Mlp1p and Mlp2p, as SUMO conjugates, and Crm1p as a SUMO direct and indirect interactor [59].

Finally, we observed that co-depletion of SUMO-1 with Tpr nearly abrogated the senescence induced by Tpr depletion. It may therefore be hypothesized that SUMO-1 depletion corrects the accumulation of free SUMO-1 in Tpr-depleted cells. In support of this, under normal conditions SUMO-1 appears to be mainly conjugated to target proteins, while unconjugated pools of SUMO 2/3 species are abundant and available. However, in contrast with SUMO 2/3 [54], SUMO-1 overexpression does not seem to have a direct effect on the process of cellular senescence [60]. Therefore, the exact contribution made by decreased SUMO-1 conjugation and an enlarged pool of free SUMO-1 to Tpr-induced senescence is not clearly understood at present, whether in conjunction with p53 or not. Up until now it was agreed that disruptions in the SUMO pathway that result in increased levels of SUMOylated proteins would trigger senescence [61], [62]. Our observations do not exclude the possibility that increased SUMO modifications contribute to the execution of the senescence program and that its knockdown precludes completion of this program. Further experiments will be necessary to clarify these issues along with the molecular mechanisms involved.

## Materials and Methods

### Cell lines and culture conditions

HeLa cells, A375, 293 and U2OS cells were obtained from ATCC. All cells were cultured in DMEM Glutamax (invitrogen), 10% fetal calf serum and 5% CO2.

### RNA interference and DNA constructs

Two siRNA Tpr sequences were designed corresponding to 141–159 nt (dsTpr2) and 1381–1399 nt (dsTpr1) of the Tpr human coding sequence (Genbank NM_003292; V. Cordes, personal communication and reference [63], respectively). DsTpr2 was used in most experiments as Tpr siRNA. Another sequence, Tpr iv [64], was tested in senescence induction (Fig. S2). Other siRNAs corresponded to Nup133 (a gift from V. Doye), Nup153 (Qiagen) and a non-targeting siRNA (Dharmacon, Eurogentec). Other siRNAs were synthesized at Dharmacon and Eurogentec. SENP2 siRNAs corresponded to sense GGUAACUUCUGCUUGUAAU·d(TT) [65] and sense GAUCAGAGUGACAGUUACC·d(TT) [66], SUMO-1 siRNA: sense CUGGGA AUGGAGGAAGAAG·d(TT) [67], p53 siRNA: sense CAGUCU ACCUCCCGCCAUA.d(TT) [68], Crm1 siRNA sense UGUGGUGAAUUGCUUAUAC·d(TT) [69].

HeLa cells were seeded in 24-well dishes at a concentration of 5 10^4^. 6 hours later, the cells were transfected with 3 µl Oligofectamine or with 1 µl Lipofectamine RNAiMax and 3 µl siRNAs (20 µM). Forty-eight hours after transfection, protein levels were analyzed by Western blot analysis or cells were fixed for immunofluorescence studies.

### Growth curves, cell cycle and apoptosis analysis

HeLa cells were seeded as described in triplicate and transfected with Tpr siRNAs, mock siRNAs or not transfected. The cells were trypsinized and counted every day after transfection.

Apoptotic cell death was determined by measuring surface exposure of phosphatidylserine. Double staining for Annexin-V-Fluorescein and propidium iodide (PI) was performed using the Annexin-V-FLUOS staining kit (Roche). Cells that stained positive for PI (dead cells) were excluded from the analysis. Data were acquired on a FACSCALIBUR cytometer and analyzed using CellQuest software (both Becton–Dickinson, Pont de Claix, France).

In the cell cycle distribution analysis, cells were harvested every day, fixed overnight in 70% ethanol, washed and incubated with 100 µg/mL RNAseA and 10 µg/mL propidium iodide in PBS for 1/2 hour at RT. Samples were analyzed for DNA content and cell size on a FACSCALIBUR cytometer using standard methods. 30 000 gated events were counted for each sample. The histogram data were curve fitted and percentage cells in each phase of the cell cycle were estimated using Modfit software.

### Clonogenic cell survival assay

HeLa cells were transfected with Tpr-specific siRNAs and mock controls in duplicate. 48 hours after transfection, cells were counted and seeded in triplicate 100 mm dishes as 100 cells per dish. The cells were then left to grow for 15 days. Colonies were stained with 0.5% crystal violet solution in 25% ethanol and counted.

### BrdU incorporation

For BrdU incorporation, cells were pulsed with 10 µmol/L BrdUrd for 14 h at 37°C. Cells were fixed in 3,2% PFA. DNA was denatured by acid treatment (2 M HCl) and BrdU was labeled with a mouse anti-BrdU-FITC antibody (BD Pharmingen™).

### Assay of the senescence-associated β-galactosidase

Cells were fixed in 2% formaldehyde/0.2% glutaraldehyde at room temperature for 5 min, and incubated at 37°C with a fresh staining solution (1 mg/ml of 5-bromo-4- chloro-3-indolyl β-D-galactoside, 40 mM citric acid-sodium phosphate (pH 6.0), 5 mM potassium ferricyanide, 5 mM potassium ferrocyanide, 150 mM NaCl, and 2 mM MgCl2) [30].

### TNF stimulation and NFκB nuclear export analysis

HeLa cells were transfected with Tpr or mock siRNAs 2 days prior to NFκB induction. Transfected and control cells were then incubated with TNF 100 IU/mL (Pharmingen) for 40 min. TNF was then removed from the medium and the cells were either fixed or kept for another 1h30 in fresh medium before fixation.

### Antibodies

The following antibodies were used: mouse 203-37 (Oncogene research) and 3B9 (Santa Cruz) and rabbit (a gift from L. Gerace) anti-Tpr, mouse (DO1) and rabbit anti-p53, goat anti-Nup133, mouse anti-p16, goat anti-Crm1, rabbit anti-NFκB (Santa Cruz), mouse anti-Nup153 (Progen and SA-1, a gift from B. Burke), mouse anti-emerin (Novacastra), mouse anti-FG nucleoporins Mab414 (Eurogentec and Covance), goat anti-RanGap1 antibody (a gift from F. Melchior), mouse anti-p21 (Calbiochem), rabbit anti-SENP2 (Abgent and Calbiochem), mouse anti-GMP-1 (anti-SUMO-1, Invitrogen), mouse anti-Rb (BD Pharmingen), rabbit anti-TRF2 (Novus biologicals), rabbit anti-histone macroH2A1 (Active Motif), mouse anti-HP1γ and mouse anti-RPB1(Euromedex), mouse anti-actin AC-40 and anti-α tubulin DM1A (SIGMA).

We were also provided with anti-Tpr rabbit polyclonal antibodies prepared by Covalab (Lyon, France) against a combination of peptides corresponding to aa 2049–2070 and aa 2324–2349 of the human Tpr coding sequence coupled to KLH.

### Immuno-fluorescence microscopy

Cells were fixed in 3,2% paraformaldehyde, permeabilized in Triton X-100 (0.4% in phosphate-buffered saline, PBS) and stained subsequently with specific antibodies at the appropriate dilution in PBST 5% milk. DAPI (SIGMA) was used to visualize the nuclear compartment. Immunofluorescence analysis was performed using a DMRX4 fluorescence microscope and images were taken with a Hamamatsu ORCAII-ER cooled CCD-camera and processed with Openlab® software (Improvision). Some images were acquired on a Zeiss Axiophot fluorescence microscope fitted with an Olympus DP71 camera running Olympus Cell∧A software for image acquisition.

The pSTAT1-NES-GFP construct encodes residues 367–427 of human STAT1, which confers NES activity [35]. HeLa cells were co-transfected with this construct and Tpr, Crm1 and control siRNAS with JetPrimeTM following the manufacturer's instructions. 48 hours later, live cells were labeled with Hoechst 33342 and directly analyzed for GFP distribution by fluorescence microscopy. The cells then were fixed with 3,2% PFA, permeabilized and then labeled with anti-Crm1 and Tpr antibodies and Alexa fluor 546-anti-goat and Alexa fluor 488-anti-mouse secondary antibodies. Nuclei were labeled with DAPI.

### Western blot analysis

Whole cell extracts were prepared in RIPA buffer (50 mM Tris, pH 8.1/150 mM NaCl/0.2% SDS/1% sodium deoxycholate/1% Nonidet P-40/5 mM EDTA) or ProteoJET™ Mammalian cell lysis reagent (Fermentas) containing proteases inhibitors (Roche) and 1 mM PMSF. Nuclear and cytoplasmic extracts were prepared using the ProteoJET™ cytoplasmic and nuclear protein extraction kit (Fermentas). In the SUMOylation analysis, 10 mM iodoacetamide were added to all buffers.

## Supporting Information

Figure S1 **Comparison of Tpr1 and Tpr2 siRNA depletion efficiency.** S1A: HeLa cells seeded as 5 10^−4^ cells per well in 24-well plates were treated with Tpr1, Tpr2 or mock siRNAs. At 24 hours (d1), 48 hours (d2) or 72 hours (d3) after treatment with the siRNAs as indicated at top of the figure, a cell extract was analyzed by western blotting using anti-Tpr Mab 203-37. Nup62 revealed by the Mab414 antibody was used as a loading control. S1B: Cell-cycle distribution in Tpr1, Tpr2 and mock siRNAs-treated HeLa cells. Cell cycle distribution in Tpr1, Tpr2 and mock siRNA-treated cells 3 and 4 days after transfection analyzed by FACS and represented as percentage of the population. S1C. Colony forming ability after Tpr depletion in HeLa cells: HeLa cells transfected with Tpr were collected on day 3 of depletion, counted and plated at different dilutions. Light gray column: mock siRNA-treated cells; dark grey column: Tpr siRNA.
(TIF)

Figure S2 **Analysis of Crm1 expression and distribution in HeLa cells by immunofluorescence.** S2A–S2B. HeLa cells were transfected with Tpr, Crm1 and mock siRNAs as indicated on the left. The cells were fixed on day 2 post-transfection and labeled with an anti-Crm1 antibody and DAPI (S2A), anti-Tpr antibody and DAPI (S2B, top panel), anti-Crm1 and DAPI (S2B, lower panel). Scale bar: 10 µm.
(TIF)

Figure S3 **Tpr depletion induces senescence in U2OS cells**. S3A. Analysis of the expression of SENP2 in nuclear extracts of 293 cells treated with Tpr, SENP2 and mock siRNAs by western blotting analysis. Nup133 is used as a loading control. S3B. U2OS cells were transfected with mock or Tpr siRNAs. Two days post-transfection, whole cell lysates were analyzed by immunoblotting using antibodies directed against SUMO-1, RanGAP1, p53, p21 as indicated on the right. Tubulin was used as loading control. S3C. Western blot analysis of whole cell protein extracts of cells treated with Tpr siRNA or mock siRNA-treated cells. Whole cell extracts were examined for Tpr, SUMO-1 conjugates and free SUMO-1 expression. Tubulin was used as loading control. S3D. Quantification of SA-β-gal-positive U2OS cells in cultures depleted of SENP2, Tpr iv and mock depleted.
(TIF)
