# Supplementary figures and images for: Silencing Nuclear Pore Protein Tpr Elicits a Senescent-Like Phenotype in Cancer Cells
Source: PLoS One. 2011 Jul 19;6(7):e22423. doi: 10.1371/journal.pone.0022423 (PMC3139644; doi:10.1371/journal.pone.0022423)

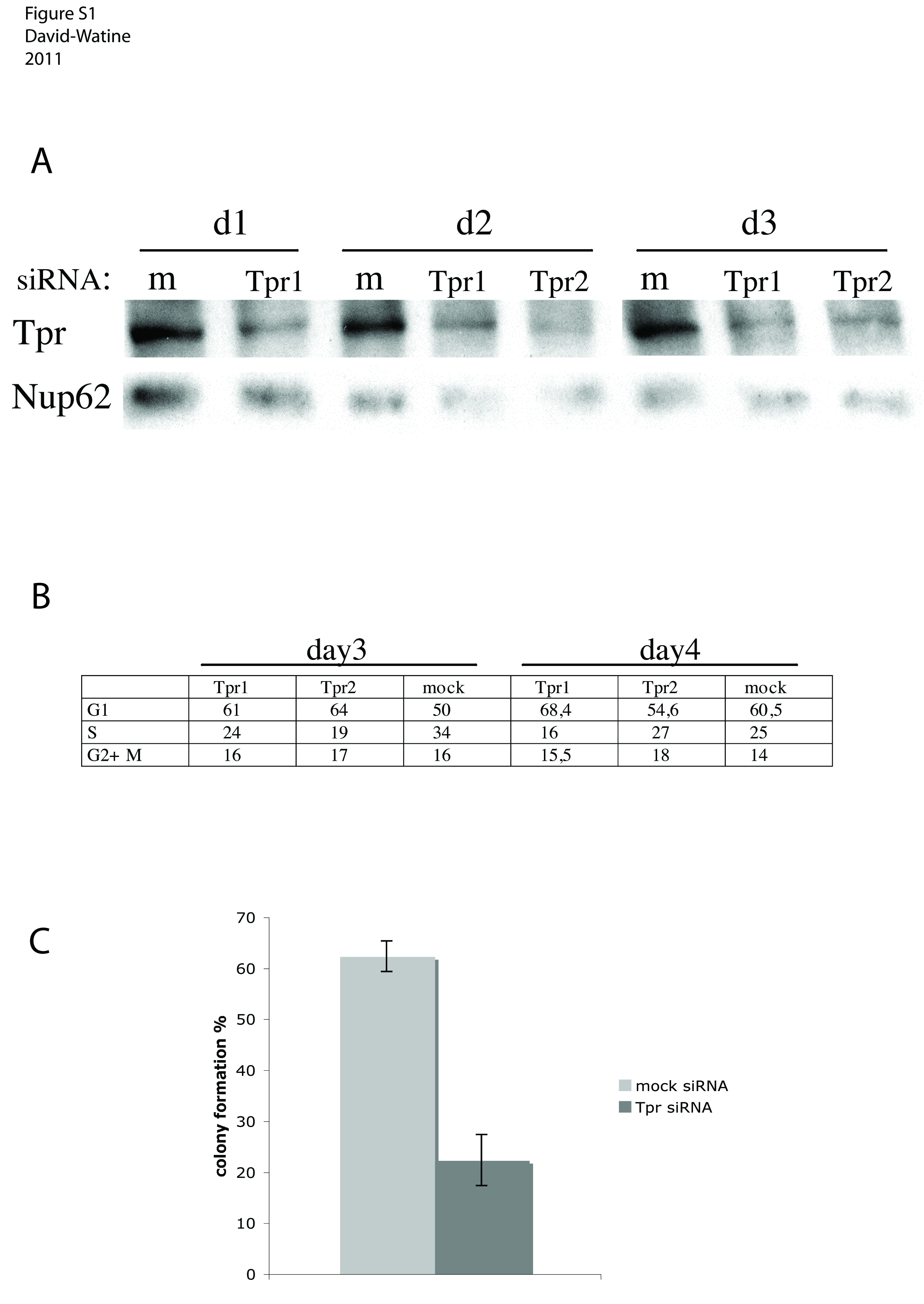

Supplement: Figure S1 — Comparison of Tpr1 and Tpr2 siRNA depletion efficiency. S1A: HeLa cells seeded as 5 10−4 cells per well in 24-well plates were treated with Tpr1, Tpr2 or mock siRNAs. At 24 hours (d1), 48 hours (d2) or 72 hours (d3) after treatment with the siRNAs as indicated at top of the figure, a cell extract was analyzed by western blotting using anti-Tpr Mab 203-37. Nup62 revealed by the Mab414 antibody was used as a loading control. S1B: Cell-cycle distribution in Tpr1, Tpr2 and mock siRNAs-treated HeLa cells. Cell cycle distribution in Tpr1, Tpr2 and mock siRNA-treated cells 3 and 4 days after transfection analyzed by FACS and represented as percentage of the population. S1C. Colony forming ability after Tpr depletion in HeLa cells: HeLa cells transfected with Tpr were collected on day 3 of depletion, counted and plated at different dilutions. Light gray column: mock siRNA-treated cells; dark grey column: Tpr siRNA. (TIF) [file pone.0022423.s001.tif]

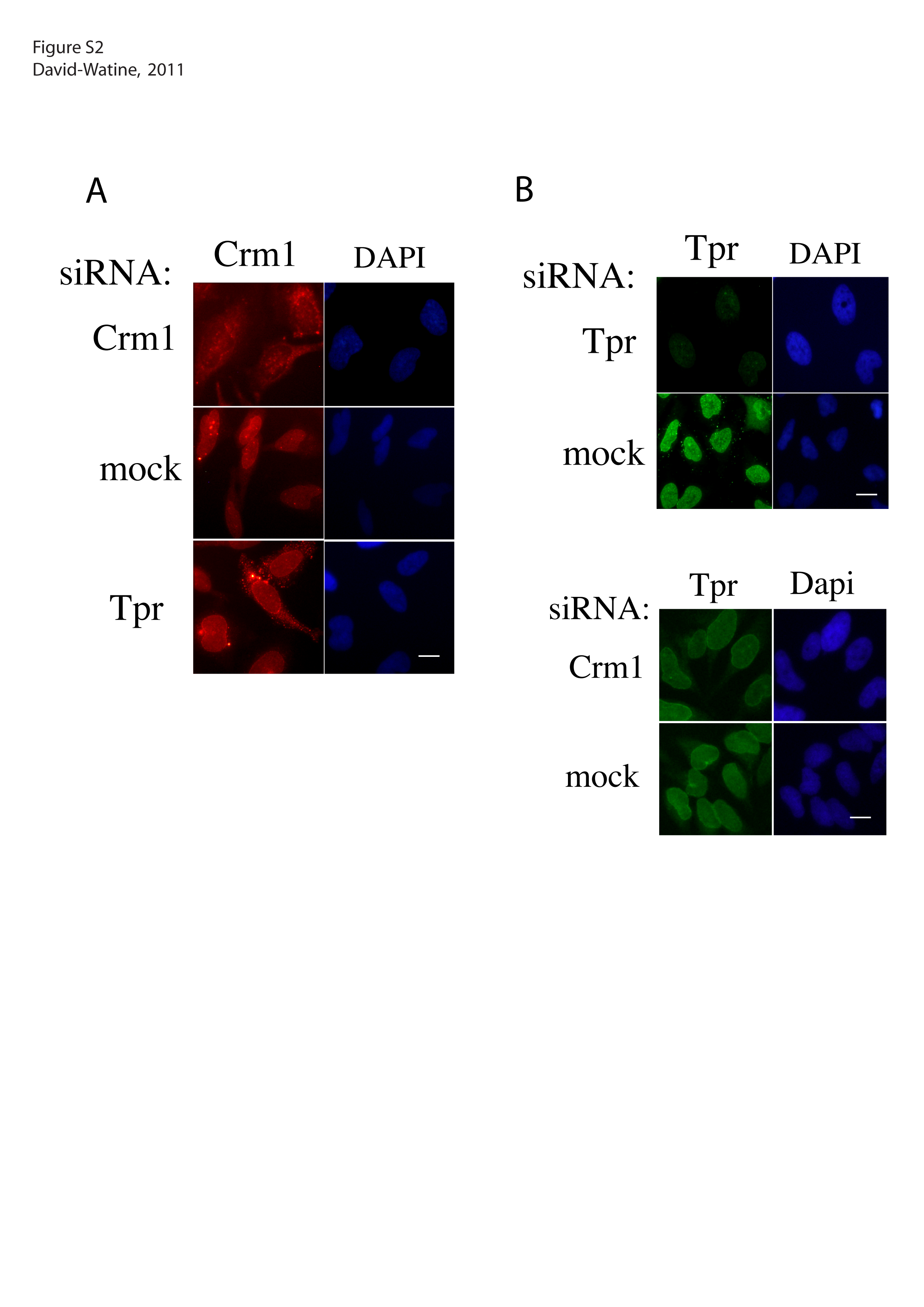

Supplement: Figure S2 — Analysis of Crm1 expression and distribution in HeLa cells by immunofluorescence. S2A–S2B. HeLa cells were transfected with Tpr, Crm1 and mock siRNAs as indicated on the left. The cells were fixed on day 2 post-transfection and labeled with an anti-Crm1 antibody and DAPI (S2A), anti-Tpr antibody and DAPI (S2B, top panel), anti-Crm1 and DAPI (S2B, lower panel). Scale bar: 10 µm. (TIF) [file pone.0022423.s002.tif]

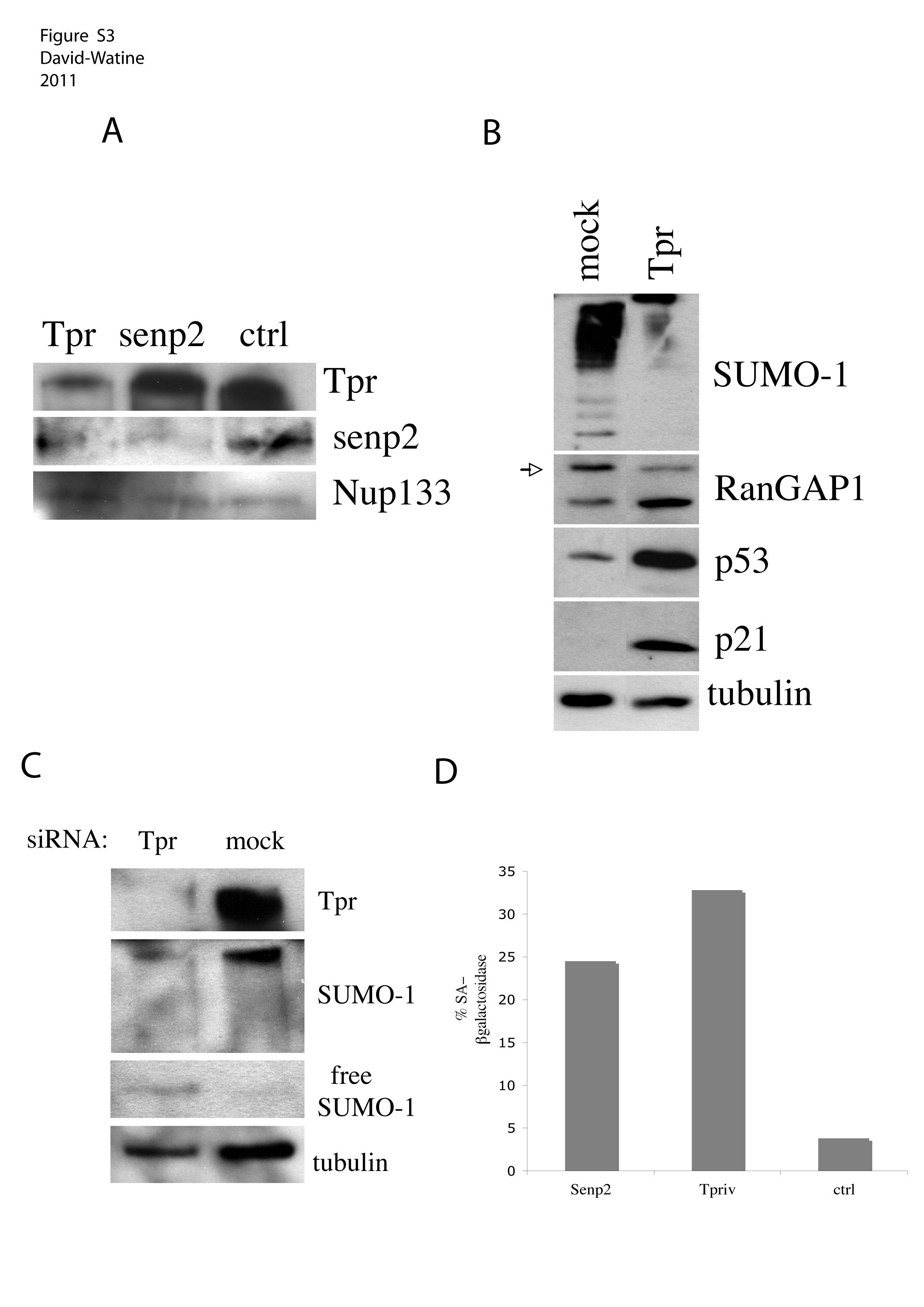

Supplement: Figure S3 — Tpr depletion induces senescence in U2OS cells. S3A. Analysis of the expression of SENP2 in nuclear extracts of 293 cells treated with Tpr, SENP2 and mock siRNAs by western blotting analysis. Nup133 is used as a loading control. S3B. U2OS cells were transfected with mock or Tpr siRNAs. Two days post-transfection, whole cell lysates were analyzed by immunoblotting using antibodies directed against SUMO-1, RanGAP1, p53, p21 as indicated on the right. Tubulin was used as loading control. S3C. Western blot analysis of whole cell protein extracts of cells treated with Tpr siRNA or mock siRNA-treated cells. Whole cell extracts were examined for Tpr, SUMO-1 conjugates and free SUMO-1 expression. Tubulin was used as loading control. S3D. Quantification of SA-β-gal-positive U2OS cells in cultures depleted of SENP2, Tpr iv and mock depleted. (TIF) [file pone.0022423.s003.tif]
